# Evaluating implementation of the FIGO Nutrition Checklist for preconception and pregnancy within the *Bukhali* trial in Soweto, South Africa

**DOI:** 10.1002/ijgo.14541

**Published:** 2023-01-12

**Authors:** Larske M. Soepnel, Catherine E. Draper, Khuthala Mabetha, Lethabo Mogashoa, Gugulethu Mabena, Fionnuala M. McAuliffe, Sarah Louise Killeen, Chandni Maria Jacob, Mark A. Hanson, Shane A. Norris

**Affiliations:** ^1^ SAMRC/Wits Developmental Pathways for Health Research Unit, Department of Pediatrics, Faculty of Health Sciences, School of Clinical Medicine University of the Witwatersrand Johannesburg South Africa; ^2^ Julius Global Health, Julius Center for Health Sciences and Primary Care University Medical Center Utrecht, Utrecht University Utrecht The Netherlands; ^3^ UCD Perinatal Research Centre, School of Medicine University College Dublin, National Maternity Hospital Dublin Ireland; ^4^ School of Human Development and Health University of Southampton Southampton UK; ^5^ NIHR Southampton Biomedical Research Centre University Hospital Southampton Southampton UK

**Keywords:** acceptability, FIGO Nutrition Checklist, mixed methods, nutrition, obesity, preconception, pregnancy, screening tool

## Abstract

**Objective:**

To evaluate implementation of the FIGO Nutrition Checklist in a low/middle‐income South African setting.

**Methods:**

This is a mixed‐methods study. Following administration of the FIGO Nutrition Checklist by a dietitian between July 2021 and May 2022, quantitative responses from pregnant (*n* = 96) and nonpregnant (*n* = 291) participants with overweight or obesity were analyzed, using logistic regression. Qualitative data from in‐depth interviews with the dietitian and a subgroup of participants (*n* = 15) were analyzed using reflexive thematic analysis.

**Results:**

Of 387 participants, 97.4% (*n* = 377) answered ‘no’ to at least one diet quality question on the FIGO Nutrition Checklist, indicative of an at‐risk dietary practice. Food insecurity was positively associated with having more than three at‐risk practices (OR 1.87; 95% CI, 1.10–3.18; *P* = 0.021). Themes from the dietitian interview included ease of use of the checklist; required adaptations to it, including explanation and translation; and benefits of the tool. Despite challenges to healthy nutrition, participant interviews identified that the checklist is acceptable and supported improved awareness of dietary intakes.

**Conclusion:**

Considering the high incidence of at‐risk dietary practices identified by the FIGO Nutrition Checklist in this population, further research into use of the tool across South African healthcare settings is warranted.

## Synopsis

The FIGO Nutrition Checklist was found to be acceptable, beneficial, and easy to implement within a dietary counselling session in South Africa.

## 1 INTRODUCTION

Optimal nutrition in the preconception period has the potential to improve women's health, pregnancy outcomes, and to reduce the noncommunicable disease (NCD) risk for their offspring.^1^ In South Africa, as many as 64% of women have overweight or obesity.^2^ In addition, South African national data from April–May 2021 indicate that 35% of households reported running out of money for food in the past month and 17% reported household hunger.^3^ These public health concerns of undernourishment, obesity/overweight, and food insecurity form a combined threat,^4^ both in the preconception period and during pregnancy. Improved nutrition is therefore a critical target for interventions to improve maternal health and to potentially reduce the risk of NCDs across generations.

The *Bukhali* randomized controlled trial, which is part of the Healthy Life Trajectories Initiative launched in South Africa, China, India, and Canada, aims to guide policy and practice around preconception health by evaluating a complex cumulative intervention, starting in the preconception period and continued through pregnancy and postpartum, in Soweto, South Africa.^5^ Within the *Bukhali* intervention, which has been described in detail previously,^6^ identification of dietary risks and behavior change are important intervention ingredients. In this study, at‐risk women in the intervention arm with either overweight or obesity attend at least one counselling session with a certified dietitian. This is based on guidelines recommending access to a dietitian in the case of obesity,^7^ since evidence suggests that support delivered by dietitians may be more effective compared with that delivered by nondietitians (including community health workers and healthcare professionals).^8^ Despite this, dietary counselling led by a dietitian is not the norm for preconception or antenatal care in South Africa, and even in high‐income countries clinical practices vary between healthcare professionals, partly due to a lack of standardized resources and training.^9^

The FIGO Nutrition Checklist was developed in 2015 as part of FIGO's initiative to improve adolescent, preconception, and maternal nutrition.^10^ The checklist was designed to be an easy to use, quick, and cross‐culturally applicable tool, which aims to identify nutritional issues and set the foundation for conversations around healthy eating behavior. Use of the FIGO Nutrition Checklist has been found to be acceptable, feasible, and valid in a clinical setting for pregnant women in Ireland^10^, ^11^ and aligned well with the results from a more laborious, validated Food Frequency Questionnaire.^12^ The high proportion of women with at least one at‐risk dietary practice in these studies (80%–95%) emphasized the importance of discussing diet with women of reproductive age.^10^, ^12^ The FIGO Nutrition Checklist was designed to be adaptable to various cultural and geographical settings, including settings such as Soweto, where sociocultural norms around obesity and overweight,^13^ limited health literacy,^14^ and food insecurity (in an estimated 33% of participants^15^) may play a role. Therefore, the FIGO Nutrition Checklist was incorporated into the *Bukhali* intervention to be used by the dietitian.

The aim of the present study was three‐fold: to evaluate the implementation of the FIGO Nutrition checklist by: (1) assessing data on at‐risk dietary practices yielded by the checklist; (2) exploring the acceptability, usability, and perceived benefits of using the checklist within a dietary counselling session, from the perspective of both a dietitian and participants; and (3) exploring contextual factors relating to implementation of the checklist as a tool for providing dietary advice.

## 2 MATERIALS AND METHODS

This adapted parallel mixed‐methods study draws on both qualitative and quantitative data from the *Bukhali* trial and process evaluation of the trial.^16^ Ethical approval was obtained from the Human Research Ethics Committee (Medical) at the University of the Witwatersrand (reference numbers M1811111 and M190449), including written informed consent from all participants before enrolment in the study.

### 2.1 Participants

The women included in this study were participants in the *Bukhali* trial. The trial is conducted in Soweto, an urban setting which has been characterized as obesogenic.^17^ Soweto is faced with structural and social factors that pose a risk to physical and mental health, including unemployment, food insecurity, limited health literacy, and traumatic events.^15^, ^17^, ^18^ Women aged 18–28 years were eligible for inclusion in *Bukhali* and were recruited using community‐based methods.^6^ Participants allocated to the intervention arm who were identified as being ‘high risk’ (body mass index [BMI] > 25 kg/m^2^) were invited to attend an individual counselling session with the trial's dietitian. In addition, a small number (*n* = 11) of participants (BMI < 25 kg/m^2^) were referred to the dietitian if they had nutrition‐related queries, wanted help changing their eating habits, or were underweight. For the quantitative analysis, we included all women who attended the dietary session between July 2021 and May 2022 (*n* = 387). The session took an average of 30 minutes, was conducted in‐person or telephonically, and aimed to support behavior change toward a healthier diet. Following their first session with the dietitian, a subsample of women was recruited telephonically, using a convenience sampling technique, to participate in an individual in‐depth interview about the session and use of the FIGO Nutrition Checklist, until data saturation was reached (*n* = 15).

### 2.2 Data collection

#### Quantitative data collection

Quantitative data were collected and managed using REDCap.^19^ During the dietary counselling session, the dietitian recorded data on participants' answers to the FIGO Nutrition Checklist questions as well as session field notes. The FIGO Nutrition Checklist includes questions about BMI, dietary requirements, and ‘yes/no’ questions on dietary quality based on a specific frequency of consumption per week for the following food groups: meat or chicken, fruit and vegetables, fish, dairy products, wholegrain carbohydrates, and packaged snacks and sugared beverages. In addition, it includes three lifestyle questions about folic acid supplement intake, sun exposure, and iron levels (Appendix S1).^20^ The question on folic acid supplementation was only asked of pregnant participants, and supplements were provided to the intervention arm of *Bukhali*.^6^ If participants answered ‘no’ to one of the six diet quality questions on the checklist, this was categorized as an ‘at‐risk dietary practice.’ Additional data collected at baseline and during the intervention by research staff are shown in Table 1.

**TABLE 1:** Detailed description of quantitative and qualitative data collection methods.

Quantitative data collection			
Variables	Measurement method	Measurement time point	Data management and calculated variables
Participant age	Self‐report (date of birth)	Dietary session	‐
Level of education	Self‐report	Baseline	‐
Obstetric history	Self‐report	Baseline	‐
Pregnancy status at time of dietary counselling session	Self‐report to health helper	Dietary session	For this study, the ‘not pregnant’ category includes participants in the postpartum phase
Weight, height	Anthropometric measurement using a SECA scale and Holtain stadiometer	Baseline and at 6, 12, and 18 months after enrolment, and, in case of pregnancy, at 10–17 weeks and 24–28 weeks gestational age. The measurement closest in time to the dietary session was used^6^	BMI (weight in kg/height in m^2^) categorized according to WHO classifications
Food security	Self‐report based on a shortened, three‐question version of the Community Childhood Hunger Identification Project (CCHIP) Index	Baseline	Categorized into ‘not food insecure,’ ‘at risk of food insecurity,’ and ‘food insecure,’ as described by Kehoe et al.^15^

Qualitative data collection		
	Dietitian interview	Participant interviews
Interviewer characteristics	Senior researcher (CAD, PhD) associated with HeLTI *Bukhali* with extensive qualitative research experience; female; English‐speaking	Project coordinator (GM) trained in qualitative data collection and familiar with the trial and local context; female; able to converse in participant home languages in addition to English
Topics covered in semi‐structured interview guide	Dietitian's experience with and perception of FIGO Nutrition Checklist: acceptability, usability, adaptation, perceived benefits, and suggestions for improvement Insights on the dietary counselling sessions: ‐ Challenges experienced in sessions and methods used to overcome these ‐ Impact of participants' pregnancy	Demographics Participants' thoughts on nutrition and health Experience with and perception of FIGO Nutrition Checklist questions and dietary session: acceptability, usability, adaptation, perceived benefits, and suggestions for improvement Experiences with dietary behavior change since session

#### Qualitative data collection

Individual in‐depth interviews were conducted in‐person at the study site with the trial dietitian and with a sample of participants. Interviews were conducted by research staff using a semistructured interview guide developed by the coauthors a priori, as reported in Table 1.^21^ Field notes were taken, and all interviews were audio‐recorded and subsequently translated into English (where necessary) and transcribed verbatim by a third‐party provider.

### 2.3 Data analysis

#### Quantitative data analysis

STATA version 17 (StataCorp LLC) was used for statistical analysis. Descriptive data are presented as number and percentage for categorical variables and as median and interquartile range for continuous variables. For logistic regression, participants were characterized as having a ‘high‐risk’ diet if the number of ‘suboptimal dietary practices’ (answering ‘no’ to a diet quality question on the FIGO Nutrition Checklist) was greater than the 50th percentile. Logistic regression was used to explore the association of a high‐risk diet with participant characteristics including age, food security, BMI, level of education, pregnancy status and history, and length of time since enrolment in the trial at dietary session.

#### Qualitative data analysis

A reflexive thematic analysis approach was adopted to analyze the qualitative data.^22^ The analysis was a combination of a deductive and inductive approach. Themes were deductively informed by an overarching framework of the United Kingdom Medical Research Council (UKMRC) guidance on process evaluations of complex interventions, specifically the components of ‘implementation,’ ‘participant response to and interaction with the intervention,’ and ‘contextual factors’ shaping the intervention and outcomes.^23^ Within the ‘implementation’ and ‘participant response and interaction’ components, acceptability, usability, adaptation, perceived benefits, and suggestions for improvement can be considered a priori themes. An inductive approach was used to recognize themes related to the context and participant perspectives that may impact implementation of the FIGO Nutrition Checklist, as a tool, not only for screening risk, but also for providing dietary advice within the dietary counselling sessions. This allowed for an in‐depth exploration of dietitian and participant experiences and perspectives in a setting with barriers to healthy behaviors for young women.^17^ Following familiarization with the data, codes were developed and applied to the transcripts using MAXQDA 2020 software (VERBI Software 2019).

## 3 RESULTS

### 3.1 Quantitative participant characteristics and FIGO Nutrition Checklist results

**FIGURE 1:**
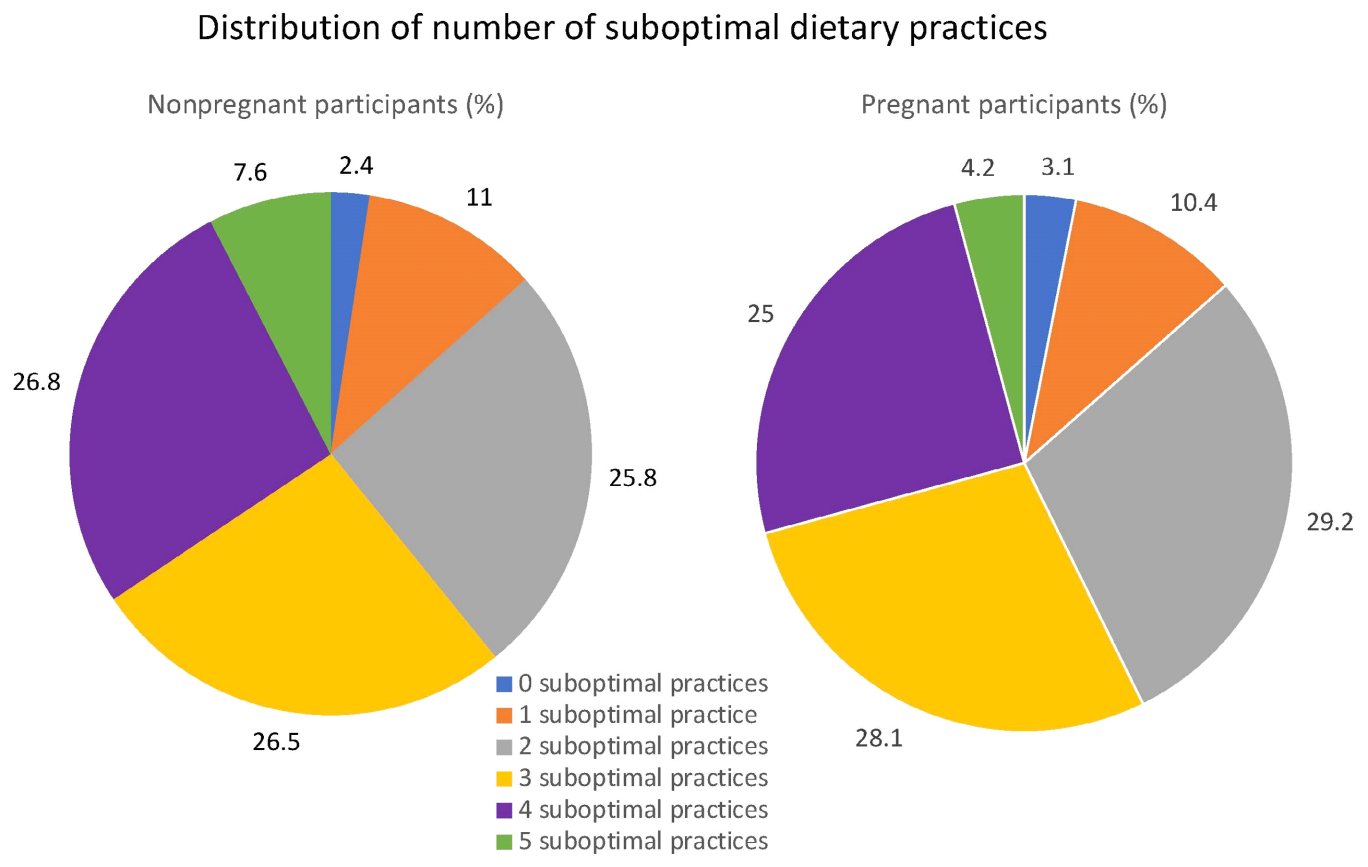
Overview of the distribution of the number of suboptimal dietary practices in nonpregnant and pregnant participants.

**FIGURE 2:**
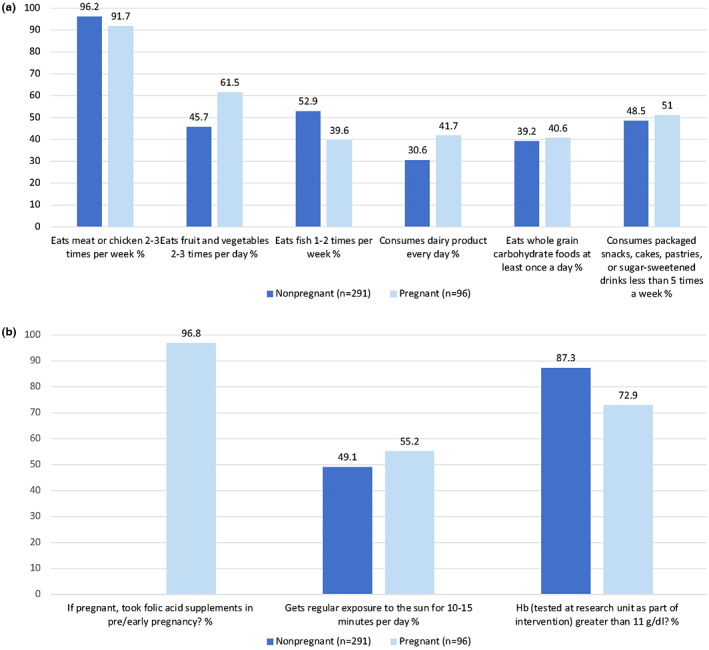
Percentage of pregnant and nonpregnant participants who reported (a) diet quality and (b) additional lifestyle practices included on the FIGO Nutrition Checklist.

Characteristics of the 387 participants attending the dietary counselling session are provided in Table 2. Of these participants, 97.4% (*n* = 377) answered ‘no’ to at least one FIGO Nutrition Checklist diet quality question, indicative of an at‐risk dietary practice, and the median number of at‐risk dietary practices was 3 (IQR 2–4) (Figure 1). While most participants reported eating meat or chicken 2–3 times per week, positive responses to the other diet‐quality questions were lower, and only 33.3% and 39.4% of participants met recommendations for dairy and wholegrain carbohydrate consumption, respectively (Figure 2).

**TABLE 2:** Characteristics of participants attending dietary support session.^a^ ^,^ ^b^

Variable	Total *n* = 387	Nonpregnant *n* = 291	Pregnant *n* = 96
Age, years	24.0 (21.8–26.3)	24.2 (21.8–26.5)	23.4 (21.4–25.9)
Time since joining intervention, months	5.2 (0.3–10.8)	6.3 (0.4–11.8)	2.1 (0.2–7.6)
Length of session, min	27 (21–35)	28.5 (21–35)	25 (20–33)
Gestational age at session, weeks	‐	‐	20.9 (13.7–29.1)^c^
BMI	32.2 (28.4–36.3)	32.8 (30.1–36.8)	29.1 (26.8–33.3)
Underweight	2 (0.5)	2 (0.7)	0 (0)
Normal weight	9 (2.3)	6 (2.1)	3 (3.1)
Overweight	109 (28.2)	60 (20.6)	49 (51.0)
Obese	267 (69.0)	223 (76.6)	44 (45.8)
Number of previous live births at baseline	^d^	^e^	
0	168 (43.6)	117 (40.5)	51 (53.2)
1	157 (40.8)	123 (42.6)	34 (35.4)
≥2	60 (15.6)	49 (17.0)	11 (11.5)
Attained high‐school diploma	216 (56.0)^f^	165 (56.9)^g^	51 (53.1)
Unemployed (excluding students)	313 (81.1)^f^	238 (82.1)^g^	75 (78.1)
Food security^h^	^f^	^g^	
Food secure	135 (35.0)	99 (34.1)	36 (37.5)
At‐risk of food insecurity	99 (25.7)	77 (26.6)	22 (22.9)
Food insecurity	152 (39.4)	114 (39.3)	38 (39.6)

Abbreviations: BMI, body mass index (categorization according to WHO classification); IQR, interquartile range.

^a^ Values are given as median (IQR) or number (percentage).

^b^ Number is out of total group unless indicated by: ^c^ *n* = 95; ^d^ *n* = 385; ^e^ *n* = 289; ^f^ *n* = 386; ^g^ *n* = 290.

^h^ Food insecurity using Community Childhood Hunger Identification Project (CCHIP) Index, with categorization as outlined in Table 1.

In logistic regression analysis exploring characteristics associated with having a high‐risk diet (>3 suboptimal dietary practices), the indicator for food insecurity was positively associated with following a high‐risk diet (OR 1.87; 95% CI, 1.10–3.18; *P* = 0.021) (Table 3). Having graduated high school (OR 0.60; 95% CI, 0.37–0.95; *P* = 0.028) and length of time since first enrolment in the *Bukhali* trial (OR 0.96; 95% CI, 0.92–0.99; *P* = 0.017) were inversely associated with a high‐risk diet. These results did not substantially change when excluding the participants attending dietary sessions who did not have overweight/obesity (*n* = 11).

**TABLE 3:** Logistic regression analysis exploring the associations between participant characteristics and having a high‐risk diet (>3 suboptimal dietary practices on the FIGO Nutrition Checklist).^a^

Dependent variable: high‐risk diet			
*n* = 384; Pseudo R^2^ = 0.046			
Variable	Odds ratio	*P* value	95% confidence interval
Age at session	0.99	0.903	0.91–1.09
Pregnant	0.72	0.231	0.42–1.24
BMI at latest measurement	1.01	0.492	0.98–1.05
Food security			
Food secure	Ref		
At risk	1.46	0.211	0.81–2.64
Food insecure	1.87	**0.021**	1.10–3.18
Live births at baseline			
0	Ref		
1	0.82	0.452	0.48–1.38
≥2	0.67	0.291	0.32–1.41
Unemployed	1.03	0.911	0.57–1.88
Graduated high school	0.60	**0.028**	0.37–0.95
Length of time (months) in trial	0.96	**0.017**	0.92–0.99

^a^ Bold text indicates statistical significance at *P* < 0.05.

### 3.2 Perceptions of FIGO Nutrition Checklist

**FIGURE 3:**
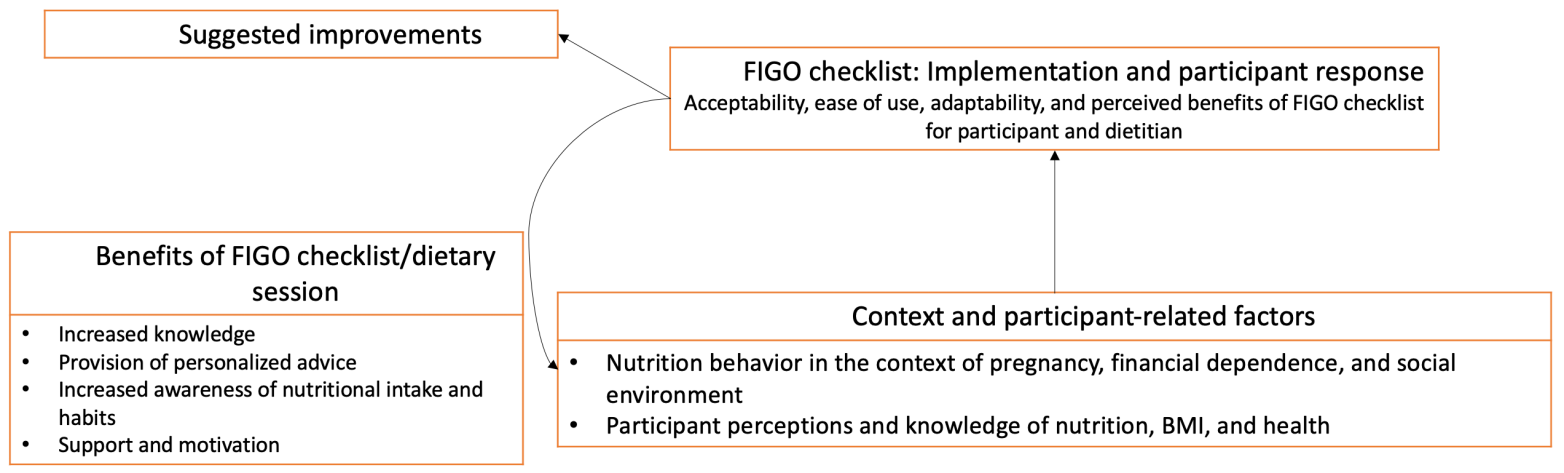
Conceptual overview of qualitative results.

**FIGURE 4:**
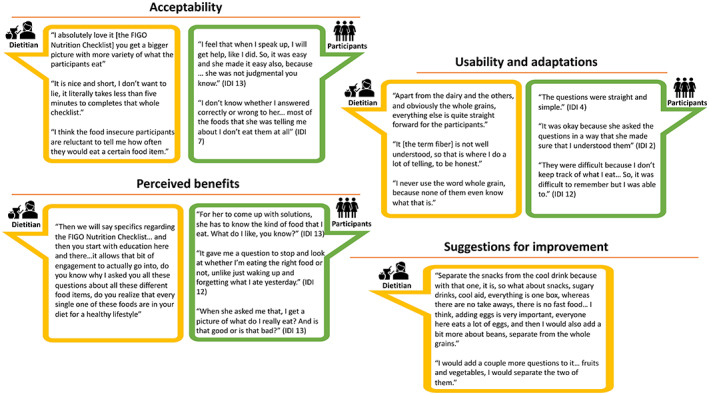
Illustrative quotations recording dietitian and participant perceptions of the FIGO Nutrition Checklist (IDI, in‐depth interview).

An overview of participant characteristics for the in‐depth interviews is provided in Table S1. A conceptual overview of the qualitative results is provided in Figure 3 and an overview of qualitative themes is provided in Table 4. Illustrative quotations on perceptions of the FIGO Nutrition Checklist are displayed in Figure 4.

**TABLE 4:** Overview of topics and qualitative themes.

Overarching topic	Source	Themes
3.2 Perceptions of the FIGO Nutrition Checklist	Dietitian	Acceptability Usability and adaptations made Perceived benefits Suggestions for improvement
	Participant	Acceptability Usability Perceived benefits
3.3 Context for the FIGO Nutrition Checklist as a tool for advice from the perspective of dietitian and participants (Table 5)	Dietitian and participant	Dietary behavior in the context of pregnancy, financial/food insecurity and social environment Participant perceptions and knowledge of diet, BMI, and health Participants' suggestions for dietary counselling sessions

#### Dietitian perceptions of the FIGO Nutrition Checklist

The dietitian found the checklist to be acceptable, fast, and efficient (Figure 4). The checklist was easy to use, but the dietitian applied some adaptations for effective use in our setting, such as translations and explanations to educate and clarify what constitutes a particular food group (in particular whole grains, fiber, and dairy). In addition, she pointed out that food‐insecure participants might feel uncomfortable answering the questions, requiring sensitivity from those administering it. When applied in this way, the dietitian felt that the checklist formed a good foundation for educating participants and giving personalized advice. The dietitian's suggestions for improvement included additions of specific foods to the checklist (such as eggs and plant‐based foods such as legumes as a source of protein) and adding the option to quantify portions per question to facilitate longitudinal follow‐up.

#### Participant perceptions of the FIGO Nutrition Checklist

Overall, participants felt comfortable answering the FIGO Nutrition Checklist questions and found them easy to answer when integrated into the dietary session as a counselling tool (Figure 4). A few participants expressed some difficulty in answering the questions due to not consuming many of the food groups or not remembering their exact intake. Participants did not express any suggestions specific to the checklist questions. One added value of the checklist was an increased awareness of participants' own dietary intake, which was perceived as a helpful step toward making healthier dietary decisions. Much like the dietitian, participants also appreciated the way the checklist facilitated more personalized diet advice based on existing practices. These benefits were in line with the participants' perceived benefits of the dietary counselling session overall, which included improved participant knowledge and awareness (a ‘wake‐up call’) and receiving support and encouragement for realistic changes. Many participants reported making changes to their diet following the session, specifically reducing portion sizes, reducing unhealthy foods, and increasing intake of vegetables and wholegrains.

### 3.3 Context for the FIGO Nutrition Checklist as a tool for advice from the perspective of dietitian and participants

Illustrative quotations per theme can be found in Table 5.

**TABLE 5:** Illustrative quotations per subtheme for section 3.3: context for FIGO Nutrition Checklist as a tool for advice from perspective of dietitian and participants

Theme	Subtheme: main findings	Illustrative quotations
3.3.1 Dietary behavior in the context of pregnancy, financial/food insecurity, and social environment	Pregnancy: Health of baby as motivation	*“What if it does something like damage the child, … like that's why I even stopped drinking cold drink, and ice because they say it's not good for me.” (IDI 15)* *“The pregnancy participants are actually more keen compared to the preconception because in this case they are more concerned about their wellbeing and the wellbeing of their babies.” (Dietitian interview)*
	Impact of cravings, changes in appetite, desire to provide nutrition	*“As pregnant women, we tend to console ourselves, that no, I will do the right thing, I will get into a good diet afterwards, now I am pregnant, my baby has to eat anything, I have to take care of my cravings and whatnot.” (IDI 1)*
		*“Yes, I think it's cravings because I'm not someone that eats a lot of sweets and I'm not someone that likes food that has salt and vinegar but now I like it.” (IDI 5)*
	Financial/food insecurity: Impact of financial and food insecurity on diet	*“It's very hard to buy healthy things that will last you the whole month. And that's why we buy a lot of starch, a lot of fats, because they last us the whole month basically…not that I am blaming my money situation, but I think it also falls into that.” (IDI 13)* *“It varies in that sometimes we do not have anything at all, sometimes there is food. So, I'm not someone that forces matters to say that even if I do not have a certain thing, I still insist upon having it.” (IDI 6)* *“The food insecure [participants] are my biggest concern because then, as part of the study, we do not give out food, and then we will tell them; “Okay, how much are you eating?”; “Once a day, this big portion”, “Okay, would it be possible for you to split it?” … and then they are like “Okay, we can try that.” But you do not push… just because someone may look a certain way, we should not judge when they say they eat one meal a day, because they might not have food at home.” (Dietitian interview)*
	Social environment: Support from family members	*“My mom and I talk and speak a lot, and she tried to prepare the food the way that [dietitian] was instructing me; since from there she's the one who prepares the food a lot lately, ja, just to make sure that I eat properly.” (IDI 7)*
	Impact of social stressors	*“My mom and I talk and speak a lot, and she tried to prepare the food the way that [dietitian] was instructing me; since from there she's the one who prepares the food a lot lately, ja, just to make sure that I eat properly.” (IDI 7)* *“Yes, you can focus on your health and your weight, but you cannot avoid other things because you want to stay healthy and take care of yourself.” (IDI 4)* *“Safety is not always guaranteed even at home. So that is one thing that is out of my control, we cannot control what is going on around my environment, you know, in terms of other people who I live with at home…you know, you are always on edge.” (IDI 13)* *“It is definitely about relationships more than anything. I think the one thing we forget is a human being holistically has so many components to them… We cry together sometimes, we cry to be honest, sometimes you just have to stop being a dietician for that moment, and be a human being, you just have to listen.” (Dietitian interview)*
3.3.2 Participant perceptions and knowledge of diet, BMI, and health	Participants' diet‐related health literacy: Lack of knowledge prior to trial participation	*“Because as young people, especially in Soweto, we do not get exposed to this kind of knowledge, you understand. So, we eat your bunny chow, like, junk food.” (IDI 1)* *“Before I joined the study, I did not know anything, I ate whatever I wanted to, now I am careful of what I eat.” (IDI 11)* *“For now, like, I found it [information] when I came here … other than that I do not have anyone that tells me about health.” (IDI 12)*
	Lack of active decision‐making around diet	*“I do not decide, I just eat what is in the fridge, you know we do not have too many choices, but we do put this and that together. What is left, we eat it.” (IDI 13)* *“I do feel some kind of way, but my heart sometimes does not want [oily food] but I do not have a choice because I have to eat and that's the food that's available.” (ID 6)*
	Participants' perception of BMI classifications and body image: Reaction to disclosure	*“My heart was shattered, I will not lie, I was shocked, like, seriously? I would always console myself and say oh well my father's side of the family are chubby, so even if I see myself gaining weight, I was like oh well.” (IDI 8)* *“Oh, the word is painful, like the word obese is painful, it gives you that mind that you are obese and I'm still so young.” (IDI 12)* *“How could you say someone in my weight is obese whereas there are bigger ones than me! So, I would not say I'm obese, I would say I'm a normal size than obese.” (IDI 4)*.
	Dichotomy between body image and advice	*“They said I should lose some weight. I told them that my weight was fine … being slender is not fun, I used to be there, I love this body…I used to be made fun of.” (IDI 4)* *“I feel like the ones who say, ‘do not even bother,’ it's not their body. They are seeing me as being beautiful but I'm the one who is suffering because I cannot walk long distance without getting tired. It's just a lot, I cannot wear some clothes because I do not have sizes or whatever; whereas they do not feel that pressure they just see me as having a nice body.” (IDI 12)* *“None of them want to lose weight, it's a handful of them …a lot of people have always seen, the larger you are, the more well‐off you family is, you are well taken care of, you have no chronic diseases …The minute some of them start to lose weight, they say ‘no, I do not want to do this.’” (Dietitian interview)*
3.3.3 Participants' suggestions for dietary counselling sessions	Preference for directive approach	*“She knows what pregnant women need to eat, she should have maybe given me a list of all the things that I should be eating, and the things that may be dangerous to my pregnancy if I eat them; then I'd know what to eat and what not to eat.” (IDI 5)* *“Maybe if she could write them down for me, that this is the good, and this is the bad… maybe just a file that has material that teaches you, just like the book you have here, but it must focus on weight loss and ingredients. It must teach you about fiber and so forth.” (IDI 9)*
	Requests for follow‐up and additional support	*“So, I think if she had called me every month then I would have that motivation… so I think it's necessary to have that motivation and have someone that will tell you to remember that you need to eat like this and like that” (IDI 12)* *“WhatsApp helps a lot, if you could update… they could have a group chat or maybe [dietitian] would WhatsApp to ask if we have exercised every day and so on. That would help.” (IDI 2)* *“Some do need professional help in case of mental health…You may sometimes give out advise whereas it is not advising that is needed but they need you to listen, and only then can they have a solution and heal in a way.” (IDI 4)*

Abbreviation: IDI, in‐depth interview.

#### Dietary behavior in the context of pregnancy, financial/food insecurity, and social environment

##### Pregnancy

Participant and dietitian responses indicated that being pregnant provided motivation to follow healthier dietary practices, for the sake of the baby's health. However, it would appear that pregnancy also introduced challenges for healthy dietary practices, particularly in later pregnancy, due to cravings, changes in appetite, and a desire to provide enough nutrition for the baby.

##### Financial/food insecurity

Financial dependence and, in particular, food insecurity reduced participants' physical opportunity for healthy food choices and dietary behavior change. The dietitian also discussed this challenge and the need to adapt advice to what participants are able to achieve in their circumstances. Some such suggestions included buying vegetables and nonperishables in bulk, consuming frequent small meals, starting food gardens, and considering plant‐based instead of animal‐based proteins.

##### Social environment

While some participants seemed to rely strongly on support from family members for healthy eating and making behavioral changes, participants also expressed how social stressors deter them from prioritizing their nutritional health. The dietitian similarly described the socioemotional challenges that participants face, and the need to set aside her role as a dietitian and offer emotional support.

#### Participant perceptions and knowledge of diet, BMI, and health

##### Participants' diet‐related health literacy

While participants showed knowledge around examples of specific practices that they considered unhealthy (such as fatty and sugar‐rich foods and drinks) and healthy (such as drinking water), a number of participants reported a lack of access to information around dietary health prior to enrolment in the *Bukhali* trial, suggesting a gap in young women's capacity for healthy dietary behaviors. This was reflected in the less evidence‐based ideas expressed by participants (such as sugared beverages reducing fertility). Potential sources of information outside the trial included local primary care clinics (during pregnancy), family members or partners, Google, social media, TV, and church.

##### Lack of active decision‐making around diet

Many participants described choosing what they eat according to whatever is available or craved in the moment, rather than through conscious planning or decision‐making. The impact of social and economic factors seems to be amplified by this perceived lack of choice.

##### Participants' perception of BMI classifications and body image

A common perception around the concepts of ‘overweight’ and ‘obesity’ was that these are extreme conditions not related to the participants themselves. Many participants recounted feelings of sadness and shock upon learning their overweight or obesity status as part of the *Bukhali* trial.

While some participants expressed dissatisfaction with their weight and had attempted to lose weight previously, others described their own and their society's preference for having a bigger body. This was at odds with weight loss resulting from advised dietary changes and, in some cases, with participants' own desire to become fitter or lose weight. The dietitian noted the importance of shifting focus of dietary counselling from losing weight to improving health.

#### Participants' suggestions for dietary counselling sessions

Responses indicated that some participants wanted a more directive approach to diet advice, such as food plans, lists of foods to avoid, more explanations, and information resources such as booklets. These requests may be indicative of participants' need for basic health information. Participants also wanted frequent follow‐up sessions for motivation, and additional types of support, such as mental health support and physical activity suggestions.

## 4 DISCUSSION

Use of the FIGO Nutrition Checklist has not been previously evaluated in an African setting. In the current study, in which 97.4% of participants reported at least one at‐risk dietary practice, use of the FIGO Nutrition Checklist was found to be acceptable and beneficial by both a dietitian and young women with overweight/obesity. Responses indicative of insufficient fruit and vegetable, whole grain, and dairy consumption were the most prevalent risks identified. In addition, we identified several context‐ and participant‐related factors that may impact implementation of the FIGO Nutrition Checklist in this setting.

The prevalence of at‐risk dietary practices in this study of women with overweight/obesity is similar to or higher than findings from previous studies using the FIGO Nutrition Checklist in Ireland (80%) and Hong Kong (95%).^10^, ^12^ A recent pilot study provided evidence for an association between early pregnancy checklist score and measures of placental development and gestational age at birth,^24^ highlighting the potential health consequences of high‐risk diets identified by the checklist. Our finding that the length of time since first enrolment in the *Bukhali* trial (OR 0.96; 95% CI, 0.92–0.99; *P* = 0.017) was inversely associated with a high‐risk diet additionally points to the FIGO Nutrition Checklist as a useful tool in assessing the impact of interventions. The majority of participants in our study did not meet recommendations around consumption of fruit and vegetables (50.4%), fish (50.4%), dairy (66.9%), and wholegrains (60.5%). Existing evidence points to an impact of each of these food groups on maternal health and fetal development.^25^ For instance, nutrients from fish have been found to have benefits for fetal neurodevelopment^26^; sufficient vegetable, fruit, calcium, and wholegrain content have been associated with reductions in maternal blood pressure^27^, ^28^; and wholegrain carbohydrates (as opposed to processed sugars) are associated with improved glycemic control.^29^

The acceptability of the checklist within our study supports previous findings from implementation of the checklist in healthcare settings with women in early pregnancy.^10^, ^11^ Additionally demonstrating acceptability for nonpregnant participants is significant because reaching women in the preconception period has the potential for large health return, with more time for interventions to take effect compared with those only initiated during pregnancy.^1^, ^30^, ^31^ In this life phase, however, couples may not prioritize health or future (unplanned) pregnancy outcomes over other matters,^5^, ^14^, ^32^ amplifying the importance of an intervention's convenience and acceptability. Therefore, the checklist may have potential for broader application in preconception care. It is important to note, however, that in our study the checklist was implemented by a qualified dietitian, who provided explanations and translations. The dietitian also reported the need for guidance and sensitivity, in particular in the case of food insecurity and when discussing BMI classifications. It remains to be explored whether a (translated) version of the FIGO Nutrition Checklist is still acceptable, easy to use, and beneficial when completed without such input from a dietitian, for example by community health workers or by participants themselves, as has been proposed in other settings.^10^

Factors such as social support and being pregnant were reported to facilitate changes in diet. At the same time, contextual factors such as food insecurity and social stressors, as well as cultural and participant‐related factors such as limited health literacy and perceptions around BMI classifications, were identified as potential barriers for the use of the FIGO Nutrition Checklist as a tool for advice. Interestingly, these results overlap with characteristics identified by quantitative regression analysis results as being associated with a high‐risk diet, such as lower education, food insecurity, and less time enrolled in the trial. These findings highlight that, if the FIGO Nutrition Checklist is to be employed not only to identify risk but also to provide advice for healthier behaviors in various global settings, it is important to consider the impact of relevant social, environmental, and structural factors on young women's ability to implement change on an individual level.^32^, ^33^ Applying learnings around the ways in which systemic and structural issues impact participants' interaction with and response to interventions is an essential step. Additional (social and mental health) support and an emphasis on dietary health education for women in our setting, for example, may help facilitate the role of the FIGO Nutrition Checklist in improving nutrition. In addition, the participants' negative perceptions of BMI classifications emphasize the importance of a nonstigmatizing approach, to avoid the deleterious (health) effects of weight stigma in antenatal and preconception care.^34^

The use of both quantitative and in‐depth qualitative data for evaluating the implementation of the checklist and exploring the implementation for both pregnant and nonpregnant young women are strengths of this study. A limitation is that the participants consisted mostly of women with overweight or obesity, which may impact the generalizability of our findings to all women of reproductive age. In addition, the extent to which these findings from a single‐center study translate to settings outside of Soweto requires further exploration.

The simplicity and free availability of the FIGO Nutrition checklist underlie its potential for use in various global settings. The acceptability, reported benefits, and ease of implementation of the FIGO Nutrition Checklist emphasize this potential in a lower‐resource setting, when used within a dietary counselling session among women with raised BMI. In addition, the high prevalence of at‐risk dietary practices in our study emphasizes the need for efforts to improve nutrition in this population. Implementation of the FIGO Nutrition Checklist in various healthcare and community settings, for both preconception and pregnancy care, warrants further research and should consider context‐related structural, cultural, and social challenges, such as food insecurity and limited health literacy.

## AUTHOR CONTRIBUTIONS

Shane A. Norris, Mark A. Hanson, Catherine E. Draper, and Larske M. Soepnel contributed to conceptualization and design of this study. Catherine E. Draper and Gugulethu Mabena contributed to data acquisition. Larske M. Soepnel, Catherine E. Draper, and Khuthala Mabetha performed data analysis. Larske M. Soepnel wrote the initial manuscript with input from all authors on analytical methods, writing, and critical review. All authors approved the final manuscript.

## CONFLICTS OF INTEREST

The authors have no conflicts of interest to declare.

## Supporting information


Appendix S1

Table S1
