# Supplementary material for: Evaluating implementation of the FIGO Nutrition Checklist for preconception and pregnancy within the Bukhali trial in Soweto, South Africa
Source: Int J Gynaecol Obstet. 2023 Jan 12;160(Suppl 1):68–79. doi: 10.1002/ijgo.14541 (PMC10107177; doi:10.1002/ijgo.14541)
Supplement: Supplementary file 1 — Appendix S1 [file IJGO-160-68-s001.pdf]

FIGO nutrition checklist for pre-pregnant/early pregnant women

FIGO International Federation of Gynecology and Obstetrics

the Global Voice for Women's Health

Good nutrition in the mother, both before and during pregnancy, is important in ensuring healthy outcomes for her and her baby. This checklist is designed for women to complete in conjunction with her health care professional in order to assess whether nutritional intake is sufficient, and provide a basis for the health care professional to advise where changes need to be made (if applicable).

For the woman to complete in conjunction with her healthcare professional:

1). Do you have any special dietary requirements (e.g. vegetarian, vegan, allergies)? If yes, please list below:

....

....

2). What is your:

a. Weight .... kgs

b. Height .... m

c. (Health care professional to complete): Divide weight in kg by height in metres then divide the answer by your height again to get your BMI.

Your BMI is .... kg/m^2

3). Quality of diet

i) Do you eat meat or chicken 2-3 times per week? **Yes / No**

ii) Do you regularly eat more than 2 – 3 portions of fruit or vegetables per day? **Yes / No**

iii) Do you eat fish at least 1-2 times per week? **Yes / No**

iv) Do you consume dairy products (such as milk, cheese, yogurt) every day? **Yes / No**

v) Do you eat whole grain carbohydrate foods (brown bread, brown pasta, brown rice or other) at least once a day? **Yes / No**

vi) Do you consume packaged snacks, cakes, pastries or sugar-sweetened drinks less than 5 times a week? **Yes / No**

4).What is your:

i) If you are pregnant, did/do you take folate/folic acid supplements in pre-pregnancy and in early pregnancy (first 12 weeks)? **Yes / No**

ii) Do you get regular exposure to the sun (face, arms and hands for at least 10-15 mins per day)? **Yes / No**

iii) Has the doctor/nurse tested your haemoglobin (level of iron in the blood)? **Yes / No**

(Health care professional to complete) If yes, is it more than 110 g/l? **Yes / No** Enter the value: ....

If you have answered No to any of the questions in section 3 or 4 your nutritional status may need to be assessed in more detail.

Additional details for health care provider:

The intention is that this document will be adapted to the context of the country in which it is being used.

1. A healthy BMI is usually considered to be between 18.5–25 Kg/m^2, although this depends on age and geographical region.

2. For women who are not pregnant, counsel on achieving a healthy weight before conceiving.

* For pregnant women provide indications for appropriate gestational weight gain according to pregravidic BMI (see right). This may vary according to local contexts.

Pre-pregnancy BMI category	Total weight gain (kg)	Rate of weight gain 2nd and 3rd trimester (kg/wk)
Underweight <18.5 kg/m^2	12.5 - 18	0.51 (0.44 – 0.58)
Normal weight 18.5-24.9 kg/m^2 weight	11.5 - 16	0.42 (0.35 – 0.50)
Overweight 25.0 – 29.9kg/m^2 weight	7-11.5	0.28 (0.23 – 0.33)
Obese >30kg/m^2	5-9	0.22 (0.17 – 0.27)

From 2009 Institute of Medicine guidelines on gestational weight gain: https://www.nationalacademies.org/hmd/~/media/Files/Report%20Files/2009/Weight-Gain-During-Pregnancy-Reexamining-the-Guidelines/Report%20Brief%20-%20Weight%20Gain%20During%20Pregnancy.pdf

3. Q 3. i. is to assess whether vitamin B12, iron and protein intake is sufficient.

4. Q 3. ii. is to assess whether intake of antioxidants, micronutrients and fibre is sufficient.

5. Q 3. iii. is to assess whether intake of omega 3 / omega 6 polyunsaturated fatty acids, vitamin D and iodine is sufficient.

6. Q 3. iv. if the patient answers No to this question, calcium supplementation should be considered.

7. Q 3. v. and vi. – if No, advice should be given to increase wholegrains and reduce processed sugar intake.

8. Q 4. i. if not taking a folate supplement suggest a folate supplement.

9. Q 4. ii. if the patient has little sun exposure or has dark skin, consider vitamin D supplementation.

10. Q 4. Iii. if Hb < 110 g/l suggest an iron supplement. This cutoff may vary according to local contexts.

11. Health care professionals should consider any foods available in their country which are considered unsafe for pregnancy.

12. As well as the questions in the questionnaire, health care professionals should assess whether any other potential unsafe aspects of the woman's lifestyle should be counselled on, such as smoking, alcohol, recreational drug use, or lack of physical exercise

Pre-pregnancy – when planning a pregnancy		
**Involved professionals**	**Assessment considerations**	**Discussion points**
• Community health workers<br>• Nutritionists<br>• Family doctors (GPs)<br>• Ob-gyns<br>• Midwives	• Diet composition<br>• Physical activity history<br>• Height, weight, BMI<br>• Obesity risk<br>- WC (+ other anthropometric measures)<br>• Anemia<br>• Risk of specific nutritional problems (low nutrient density)<br>- Folate<br>- Iron<br>- Calcium<br>- Vitamin B12<br>- Vitamin D<br>- Iodine<br>- Zinc<br>- PUFAs	• Importance of a healthy diet and exercise<br>• Problems of sedentary behaviour such as screen-time<br>• Risky behaviors and exposures<br>- Tobacco, alcohol recreational drugs<br>- Environmental toxins<br>• Chronic disease screening and management<br>• Supplementation<br>- Folic acid supplementation 400 μ/day<br>- Other nutrients (iron, iodine, vitamin B12)

During pregnancy		
**Involved professionals**	**Assessment considerations**	**Discussion points**
• Community health workers<br>• Nutritionists<br>• Family doctors (GPs)<br>• Ob-gyns<br>• Midwives	• Diet composition<br>• Physical activity history<br>• Height, weight, BMI, WC (other anthropometric measures?);<br>• Gestational weight gain<br>• Blood pressure<br>• Risk of specific nutritional problems (low nutrient density), deficiencies from specific diets or under-nutrition)<br>*First trimester*<br>- Folate<br>- Vitamin B12<br>- Iodine<br>- PUFAs<br>*Second and third trimesters*<br>- Iron, iodine, zinc, copper, calcium<br>- Folate, B vitamins, vitamin D<br>- Energy (+450 kcal/day)<br>- Iodine<br>- PUFAs	• Dietary counselling<br>• Safe levels of exercise<br>• Sedentary time<br>• Weight management and gestational weight gain<br>• Risky behaviors and exposures<br>- Tobacco, alcohol recreational drugs<br>- Sources of food borne infection<br>• Pregnancy complication screening and management (GDM, blood pressure)<br>• Supplementation<br>- Folic acid supplementation 400 μ/day<br>- Iron supplementation 30-60 mg/day<br>- Other nutrients as required (iodine, vitamin B12, vitamin D)

This document, including the above table, is based upon The International Federation of Gynecology and Obstetrics recommendations on adolescent, preconception, and maternal nutrition: "Think Nutrition First", Hanson M et al, 2015, which can be accessed at http://obgyn.onlinelibrary.wiley.com/hub/issue/10.1002/ijgo.2015.131.issue-S4/

FIGO is a non-governmental organisation specialising in ensuring improvement in women's health around the world. It is the only global organisation representing national societies of obstetricians and gynecologists, and works together with other medical professionals and organisations to ensure the best possible health for the women of the world.
