# Supplementary material for: Evaluating implementation of the FIGO Nutrition Checklist for preconception and pregnancy within the Bukhali trial in Soweto, South Africa
Source: Int J Gynaecol Obstet. 2023 Jan 12;160(Suppl 1):68–79. doi: 10.1002/ijgo.14541 (PMC10107177; doi:10.1002/ijgo.14541)
Supplement: Supplementary file 2 — Table S1 [file IJGO-160-68-s002.docx]

**Supporting information Table 1.** Characteristics of participants participating in the in-depth interviews

| Participant number | Age range, years | Pregnant during dietitian session | Number of children | Living with | Gestational age at time of in-depth interview (weeks + days) |
| --- | --- | --- | --- | --- | --- |
| 1 | 23-24 | Yes | 0 | Sister, Sister’s two children | 33 + 2 |
| 2 | 25-26 | No | 2 | Participant’s children, Grandfather, Sister | - |
| 3 | 23-24 | Yes | 1 | Participant’s child, Partner | 23 + 6 |
| 4 | 23-24 | No | 1 | Participant’s child, Mother, Brother, Sister | - |
| 5 | 19-20 | Yes | 0 | Participant’s children, Partner | 30 |
| 6 | 23-24 | Yes | 1 | Participant’s child, Mother, Sister, Sisters’ child | 30 |
| 7 | 21-22 | Yes | 1 | Participant’s child, Mother, Sister, Brother | 41 |
| 8 | 25-26 | No | 1 | Participant’s child, Mother, Aunt | - |
| 9 | 27-28 | No | 3 | Participant’s children, Grandmother, Siblings (x7) | - |
| 10 | 19-20 | Yes | 1 | Mother, Brother | 13 |
| 11 | 27-28 | No | 2 | Participant’s children, Mother, Grandmother, Siblings | - |
| 12 | 23-24 | No | 3 | Participant’s children, Partner | - |
| 13 | 21-22 | No | 1 | Participant’s child, Mother, Sister, Sister’s children | - |
| 14 | 19-20 | Yes | 0 | Grandfather, Aunt, Uncle, Cousins (x2) | 26 |
| 15 | 23-24 | Yes | 0 | Mother, Father, Siblings (x2), Sibling’s children | 26 |
